# Analysis of Kernel Matrices via the von Neumann Entropy and Its Relation to RVM Performances

**DOI:** 10.3390/e25010154

**Published:** 2023-01-12

**Authors:** Lluís A. Belanche-Muñoz, Małgorzata Wiejacha

**Affiliations:** 1 Department of Computer Science, Universitat Politècnica de Catalunya, 08034 Barcelona, Catalonia, Spain; 2 Cien.ai, Ronda Carrer de Sagués, 45, 08021 Barcelona, Catalonia, Spain

**Keywords:** von Neumann entropy, relevance vector machines, generalization error

## Abstract

Kernel methods have played a major role in the last two decades in the modeling and visualization of complex problems in data science. The choice of kernel function remains an open research area and the reasons why some kernels perform better than others are not yet understood. Moreover, the high computational costs of kernel-based methods make it extremely inefficient to use standard model selection methods, such as cross-validation, creating a need for careful kernel design and parameter choice. These reasons justify the prior analyses of kernel matrices, i.e., mathematical objects generated by the kernel functions. This paper explores these topics from an entropic standpoint for the case of kernelized relevance vector machines (RVMs), pinpointing desirable properties of kernel matrices that increase the likelihood of obtaining good model performances in terms of generalization power, as well as relate these properties to the model’s fitting ability. We also derive a heuristic for achieving close-to-optimal modeling results while keeping the computational costs low, thus providing a recipe for efficient analysis when processing resources are limited.

## 1. Introduction

By extending a wide spectrum of machine learning algorithms designed to operate in linear spaces, kernel methods have gained general recognition and have been applied in a variety of problems, ranging from regression and classification to principal component analysis. Given a valid kernel function, the processing of virtually all data types becomes feasible, introducing (near) infinite flexibility. The possibility of learning a suitable kernel function from the training data is another asset of a kernel method.

However, these qualities come at a cost. The performances of kernel methods tend to largely depend on the choices of the external parameters, such as the regularization (costs) as well as the kernel parameters themselves. These parameters must, therefore, be tuned by choosing them based on the model’s performance on a validation set or in cross-validation, which often proves to be extremely computationally costly. The need arises for a theoretical analysis of the implications of the kernel parameter choice with regard to the fitting ability and performance of specific kernel-based methods.

The objectives of this paper are the following: first, to understand several kernel matrix properties and the influences of kernel parameters on matrix formation and behavior; second, to derive an efficient way to avoid costly model selection techniques by providing a heuristic for the optimal model in terms of its learning ability. Since the focus of this work is placed on the choice of the kernel parameter, modifications of the support vector machine and relevance vector machine were used since they do not require additional parameters, such as the cost parameter or the epsilon insensitivity parameter. The details of this approach are discussed further in this study. This is accomplished as follows:

1. We explore the spectrum of a kernel matrix to determine the desired kernel matrix properties from the point of view of the von Neumann entropy. For that, the focus is placed on the distribution of eigenvalues and the matrix condition number. These are related to the effect of normalizing the kernel matrix and to the main contribution of this work: leveraging the understanding of the fitting ability and generalization power of the RVM models for the optimal model selection.
2. Linking the von Neumann entropy of a kernel matrix to the training error of an RVM (and, thus, its fitting ability), both for the RBF kernel and polynomial kernels, for which the fitting ability is known to be dependent on the parameters of these kernels.

Since (for many other kernel functions) such a relationship may not be known, the von Neumann entropy of a kernel matrix generated by a non-standard kernel function can be an indication of the fitting ability of a particular kernel function depending on its parameters. In this way, a kernel matrix can be qualified prior to modeling, which in turn can eliminate the risk of training a model with a kernel matrix built with parameters that yield poor performance due to not being able to fit the regularities in the data (underfitting).

### 1.1. Relevant Work

One major drawback of kernel-based methods is their sensitivity to hyperparameters that force the modeler to perform an expensive grid search often across multiple parameters. In the case of ϵ-based support vector regression, as an example, two parameters: regularizing constant *C* and width ϵ must be optimized, but kernelization of this algorithm requires choosing additional kernel parameters, such as σ for the radial basis kernel, and the cost can be prohibitive, see [1]. In 2000, Michael Tipping published his work on relevance vector machines (see [2]), introducing the RVM as a way of dealing with the SVM shortcomings. Admitting the SVM’s outstanding generalization abilities, he pointed out that it lacks the possibility to obtain a probabilistic output, i.e., classifications are ‘hard’ and predictions are point estimates.

Therefore, it is not possible to incorporate the uncertainty in the predictions in the in the form of a conditional distribution p(y|x). Furthermore, fitting an SVM requires optimizing the cost regularization parameter that controls the error–margin trade-off, and for regression—the insensitivity parameter ϵ, creating a need for expensive parameter selection techniques, such as cross-validation.

Tipping proposed a new model based on Bayesian inference, which had the same functional form as the SVM: the *relevance vector machine*. His approach included introducing a *prior* over the model weights and associating a hyperparameter with each weight that had its most probable value subsequently iteratively estimated from the data. Since in practice for many of these weights, their parameter posterior distributions exhibit peaks at around zero, the model is sparse. The vectors corresponding to the remaining weights are called *relevance vectors*.

In any event, the author mentions the limitations of his solution: the complexity of the training phase related to repeated computations and inversions of the Hessian matrix that form a part of the RVM, which can lead to the RVM being significantly slower than the SVM. In this paper, the RVM is used instead of the SVM in order to avoid any kind of dependency on the non-kernel parameters: the cost parameter *C* and the insensitivity parameter ϵ.

Even so, the kernel function parameters must still be optimized, and to address this issue, a heuristic for the choice of the appropriate kernel parameter is needed, which could be applied before the modeling starts and independently of the prediction itself. Analyzing kernel matrix properties and establishing a link between these properties and the kernel method’s performance is a promising field of study that aims to give kernel-based methods additional advantages and overcome their drawbacks.

One way of approaching this topic is to design a kernel function that does not require any additional parameter tuning. This idea was explored in [3]; the authors came up with a new extreme-learning inspired kernel function, *asymptotic ELM kernel*, which includes a σ parameter that does not further impact the performance of a support vector machine given a sufficiently large value for this parameter is provided. This kernel function is defined as follows:

(1) $$k\left(x,x^{\prime }\right)=\frac{2}{\pi }\arcsin\left(\frac{1+\langle x,x^{\prime }\rangle }{\sqrt{\left(\frac{1}{2\sigma^{2}}+1+\langle x,x\rangle \right)\left(\frac{1}{2\sigma^{2}}+1+\langle x^{\prime },x^{\prime }\rangle \right)}}\right)$$

where *x*, $x^{\prime }$ are vectors in $R^{d},d\in N,\;\sigma \neq 0$ and $\langle x,x^{\prime }\rangle$ stands for the dot product. The authors state that their results are equally good or even better than the results obtained using a Gaussian kernel. They conclude that the proposed kernel is indeed parameter-insensitive and it is sufficient to choose a large enough value of σ to guarantee error rates comparable to the ones output by the Gaussian kernel using the value of σ parameter optimized in cross-validation.

Several measures of the kernel matrix quality were proposed, such as the *regularized risk* in [4], *hyperkernels* in [5], and *kernel-target alignment* [6]. However, the first two measures require using a costly optimization technique to solve a quadratic optimization problem, which is not the desired property given the already high computational complexity of the kernel SVM. The latter is the most widespread kernel learning method and was introduced as a measure of similarity between a kernel and a target, or between two kernels. It is defined for kernel functions $k_{1}$ and $k_{2}$, represented by the corresponding matrices $K_{1}$ and $K_{2}$, as follows:

(2) $$KTA\left(S,k_{1},k_{2}\right)=\frac{{\langle K_{1},K_{2}\rangle }_{F}}{\sqrt{{\langle K_{1},K_{1}\rangle }_{F}{\langle K_{2},K_{2}\rangle }_{F}}}$$

where *S* is the data sample and *F* denotes the Frobenius inner product between matrices defined as:

(3) $${\langle K_{1},K_{2}\rangle }_{F}=\sum_{i,j=1}^{n}K_{1}\left(i,j\right)K_{2}\left(i,j\right)$$

KTA is capable of capturing to what extent the kernel function agrees with the learning task in question and whether—given a data sample—the most suitable kernel function can be estimated. The authors of this approach argue that an essential factor determining the model’s performance is the extent to which the kernel function fits the learning target and, thus, they postulate that the data distribution in the feature space should be in a way correlated to the label distribution.

However, the authors of [7] point out an important drawback of KTA learning: obtaining high estimated alignment values is a sufficient condition for a kernel matrix to be ‘good’, but it is not a necessary condition. This means that a well-behaved kernel matrix can have a very low alignment value due to the fact that KTA imposes upper bounds on the alignment value for some kernel functions and may underestimate the quality of some popular kernel functions, such as the Gaussian kernel.

To address these issues, the authors propose a *feature space-based kernel matrix evaluation measure (FSM)* that uses data distributions in the feature space and measures the within-class and between-class spread in the data for a classification task. The details of this approach can be found in [7].

An alternative way of addressing the task of parameter selection for kernels is treating the problem as a supervised learning task. In [8,9,10], *meta-learning* is applied for SVM parameter selection. As described by the authors, in the meta-learning process each so-called meta-example, i.e., a learning problem, ‘remembers’ and stores the meta-data about the corresponding problem as well as the performance of the model when using different sets of parameters. Based on that knowledge the meta-learner subsequently aims at predicting the best set of parameters for a new problem given its meta-data, i.e., characteristics of the problem, assuming that learning problems with similar characteristics yield good performance for a similar configuration of parameters.

Moreover, the meta-learner is able to rank parameter configurations so that if the first recommended set of parameters does not give sufficiently good results, subsequent recommendations can be explored. In this setting, the choice of optimal parameters for a machine learning model is a supervised learning problem itself and is, therefore, also called *learning to learn*. The topic has been explored even more thoroughly in [11] where a new method is introduced that combines meta-learning and standard search techniques for SVM parameter selection.

Furthermore, in [12], a comprehensive evaluation of the performance measures for tuning SVM parameters is presented. The study showed that with k-fold cross-validation as a benchmark that gives very good estimates of the generalization error, measures, such as *Xi-Alpha bound* and *general approximate cross-validation (GACV)* may perform relatively well. Using Xi-Alpha results in the test error close to the actual minimum but the estimated hyperparameters are often not close to the optimal ones. GACV, on the other hand, moves closer to the optimal parameters and correlates better with the test error; however, this behavior is only observed on some datasets. The rest of the measures explored by the authors did not prove useful.

### 1.2. von Neumann Entropy

Entropy is a state function of fundamental importance in physics, information theory, and various other sciences. The *von Neumann entropy* is an extension of the standard Shannon entropy to positive definite matrices, as described e.g., in [13,14,15] and defined as:

(4) E(K)=−tr(KlogK),K⪰0,tr(K)=1

where $K_{n\times n}$ is a kernel matrix with trace 1 and n∈N is the number of rows in the dataset, ⪰ denotes that *K* is positive semi-definite and log denotes matrix logarithm operation with base 2. It was shown in [14] that von Neumann entropy can also be calculated as the Shannon entropy of the matrix eigenvalues:

(5) $$E\left(K\right)=-\sum_{i}\lambda_{i}\log\lambda_{i}\;$$

where i∈{1,…,d}, *d* is the data dimension, and $\lambda_{i}$ are the eigenvalues of the matrix *K*. This is one of the most used functions today for measuring uncertainty about the state of a quantum system, in which it is commonly written as $S\left(\hat{p}\right)=-k_{B}tr\left(\hat{p}\ln\hat{p}\right)$, being $\hat{p}$ a density operator, describing the state of the system, and $k_{B}$ Boltzmann’s constant—see [16] for a modern introduction.

Since it is related to the eigenvalues of a matrix, the notion of entropy in a system with a certain probability distribution can be extended to the distribution of eigenvalues in the context of linear algebra. In such a setting, matrix eigenvalues can be transformed by dividing them by their sum and such a set of normalized eigenvalues can be treated as a probability distribution.

It is worth noting that matrix eigenvalues are used in data science as a measure of the information amount in the data in the principal component analysis. This method relies on the fact that large eigenvalues reflect a large spread in the data along the direction indicated by the corresponding eigenvector [17], i.e., if only the first few eigenvalues of a data matrix are significantly large, the dimensionality of the dataset can be reduced to these first few dimensions without a significant loss of information due to the low spread of the data on the remaining dimensions.

Maximum entropy methods have been applied to a variety of problems in the field of quantum mechanics [18] and spectral clustering [19], but the authors of [13] generalized the notion of maximum entropy to kernel matrices. They estimated a kernel matrix by maximizing the von Neumann entropy subject to a set of constraints and identified the diffusion kernel [20,21] as the one maximizing the entropy. However, it turned out that although the diffusion kernel tends to perform very well when used with a support vector machine for prediction, it has scaling problems in the feature space. The authors addressed this issue by imposing local constraints on the kernel matrix instead of global ones and consequently improved the prediction quality.

The von Neumann entropy has recently found applications in [22] to weigh kernels when integrating heterogeneous biological data from various sources and combining the different sources of information into a unique kernel. More recent uses are related to network analysis [23] and as a spectral complexity measure [24].

## 2. Conceptual and Numerical Analysis

### 2.1. Influence of the Kernel Parameter on the von Neumann Entropy

In order to understand the relationship between the von Neumann entropy and the kernel method’s performance, the behavior of the von Neumann entropy itself must be explored. To obtain a relative measure of the von Neumann entropy, which could be compared across matrices of different dimensions, in each case, the von Neumann entropy is normalized by the maximum von Neumann entropy, i.e., $\log_{2}n$ where *n* is the dimensionality of the analyzed kernel matrix. The obtained measure is a *relative von Neumann entropy* that takes values between 0 and 1, and from now on will simply be referred to as the von Neumann entropy.

Moreover, the definition of the von Neumann entropy in the quantum physics setting includes an assumption that the matrix has trace 1, which guarantees that its set of eigenvalues can be viewed in a probabilistic way since all the eigenvalues are between 0 and 1 and they sum up to 1. For that reason, when computing the von Neumann entropy of a matrix, the eigenvalues are divided by their sum.

Extreme values of the von Neumann entropy are reached for extreme forms of the matrix. The identity matrix $I_{n}$ represents the case when the amount of information is the highest. This is reflected in the fact that all of its eigenvalues are equal to 1 and, thus, the distribution obtained by dividing each eigenvalue by the sum of the eigenvalues is uniform.

We can analyze this setting by making an analogy to the probability mass distribution as discussed when the notion of entropy was introduced. If we do so, the probability of each state in the resulting probability mass distribution is equal and therefore the entropy of the system is maximum. This is analogous to the fair coin toss case. Thus, the closer a matrix is to the identity matrix, the higher its von Neumann entropy.

In the other extreme, a matrix consisting of 1s exclusively, denoted $J_{n}$, brings the least information possible as its eigenvalues are 0 apart from the first one that equals 1. When converting the set of eigenvalues into a probability mass distribution, there is only one state with non-zero probability and its corresponding probability is equal to 1. Such a system is fully predictable and there is no uncertainty at all related to its behavior; thus, its von Neumann entropy is 0. Due to the continuity of the von Neumann entropy as a function of matrix entries, between these two extreme cases, the von Neumann entropy takes values from 0 to 1 depending on the matrix spectrum. This continuity will be elaborated upon further in the study.

The radial basis function kernel is defined as:

(6) $$K\left(x,x^{\prime }\right)=\exp\left(-\sigma ∥x-x^{\prime }{∥}^{2}\right)$$

the width σ>0 of the kernel controls the flexibility of the kernel machine. It is the inverse of double the width in the Gaussian distribution used in the evaluation of the kernel function of two data points and it amplifies the distance between these points.

Therefore, for very small values of σ the value of the kernel function tends to 1 regardless of the data points it is evaluated on. This leads to underfitting the data since ‘everything is similar to everything’ according to the kernel function in this case and the resulting model has high bias and low variance. The resulting kernel matrix is close to the matrix of 1 s and *J*; thus, its von Neumann entropy tends to 0.

In the opposite case, when values of σ are very large, the kernel expression evaluates to values very close to 0 for all pairs of data points apart from the diagonal elements of the kernel matrix where it equals to 1. The model becomes more local and tends to overfit the data since in this case ‘everything is dissimilar to everything else’, the data sample can be fit perfectly due to the infinite flexibility of the model and the kernel matrix is close to the identity matrix *I* leading to high variance and low bias. Thus, the matrix von Neumann entropy tends to 1.

Consequently, there is a strong relationship between the value of the RBF kernel width σ, the model’s fitting ability, and the von Neumann entropy of the kernel matrix. A similar link can be established for another common kernel function, the polynomial kernel in the form:

(7) $$K\left(x,x^{\prime }\right)={\left(\langle x,x^{\prime }\rangle +c\right)}^{d}$$

Along with the increase in the degree of the polynomial, the flexibility of the decision boundary and the fitting ability of the model grow. Therefore, the risk of overfitting arises. The lowest valid degree for the polynomial kernel is d=1 and *d* can grow to infinity.

If the data rows are scaled (i.e., rows of the matrix built from the explanatory variables, i.e., the input for kernel matrix construction) to unit length and the kernel matrix is normalized, the behavior of the polynomial kernel becomes analogical to the behavior of the RBF kernel. The effect of normalizing the kernel matrix will be studied further in this work, but for now, the notion of kernel matrix normalization is introduced as [25]:

(8) $$K_{n}\left(x,x^{\prime }\right)=\frac{K(x,x^{\prime })}{\sqrt{K(x,x)}\sqrt{K(x^{\prime },x^{\prime })}}$$

After normalizing, the polynomial kernel expression becomes:

(9) $$K_{n}\left(x,x^{\prime }\right)=\frac{{\left(\langle x,x^{\prime }\rangle +c\right)}^{d}}{\sqrt{{\left(\langle x,x\rangle +c\right)}^{d}}\sqrt{{\left(\langle x^{\prime },x^{\prime }\rangle +c\right)}^{d}}}$$

and if the data rows are of unit length:

(10) $$K_{n}\left(x,x^{\prime }\right)=\frac{{\left(\langle x,x^{\prime }\rangle +c\right)}^{d}}{{\left(1+c\right)}^{d}}={\left(\frac{\langle x,x^{\prime }\rangle +c}{1+c}\right)}^{d}$$

Since $\langle x,x^{\prime }\rangle$ takes values between −1 and 1, the expression ${\left(\langle x,x^{\prime }\rangle +c\right)}^{d}$ takes values between c−1 and c+1, only taking the value of 1 for the diagonal elements of the kernel matrix. Moreover, since c≥0, if $\langle x,x^{\prime }\rangle =-1$:

(11) $$-1\leq 1-\frac{2}{c+1}\leq \frac{c-1}{c+1}=\frac{\langle x,x^{\prime }\rangle +c}{1+c}$$

and if $\langle x,x^{\prime }\rangle =1$:

(12) $$\frac{\langle x,x^{\prime }\rangle +c}{1+c}=1$$

therefore:

(13) $$-1\leq \frac{\langle x,x^{\prime }\rangle +c}{1+c}\leq 1$$

Thus, expression (10) takes values between −1 and 1, and with the increase of the degree *d* from 1 to infinity, the numerator of this expression tends to 0. This means that the kernel values tend to 0 outside of the diagonal of the matrix, leading to the formation of an identity matrix *I*. Similarly to the RBF kernel, this case represents the situation when ‘everything is dissimilar to everything else’ and the von Neumann entropy of the kernel matrix is maximum, i.e., 1.

The other extreme, the *J* matrix of 1 s, cannot be reached since the lowest valid degree is 1, but the lowest von Neumann entropy is reached for degree d=1. This value is data-dependent and is the dataset-specific lower bound for von Neumann entropy. Although the polynomial kernel only satisfies Mercer’s condition for degrees that are positive integers [26] and, thus, degree values between 0 and 1 do not generate valid kernels (since they do not guarantee positive semi-definiteness required by the kernel function definition), mathematical analysis of the behavior of the expression can be performed for degrees lower than 1.

Since (13) takes values between −1 and 1, raising it to a power d∈(0,1) will yield values greater than for d=1 and the values will increase with the decrease of *d* from 1 to 0, reaching the value of 1 in the limit d→0. This way the other extreme analogous to the RBF kernel case is reached for *d* values approaching 0: the kernel matrix tends to the *J* matrix of 1 s.

This case corresponds to underfitting the data since the kernel expression evaluates to values close to 1 for each pair of data points and ‘everything becomes similar to everything, according to the kernel function. The resulting model has high bias and low variance and the von Neumann entropy of the kernel matrix tends to 0.

A summary of the observations described in this section is presented in Table 1.

### 2.2. Eigenvalue Distribution Influence on the von Neumann Entropy

As mentioned previously, the set of eigenvalues of a matrix can be treated as a probabilistic distribution when normalizing them by their sum. This way an analogy can be made with a regular probability distribution and the standard information theory notion of entropy can be referred to.

High entropy of a system is achieved when there is a high level of uncertainty associated with it, i.e., a lot of information is gained when learning the outcome of an experiment. This, in turn, cannot be achieved when there are few significantly positive probabilities in the probability distribution. When most probability values are effectively zero, the amount of uncertainty in the system is relatively low since the outcome of an experiment can be predicted more easily.

Therefore, when aiming at maximizing entropy, the goal should be to obtain a balanced probability distribution with as many positive values of probability as possible. Then, when treating the set of eigenvalues as a distribution and using the von Neumann entropy as the Shannon entropy counterpart for matrices, the desired properties of the spectrum can be pinpointed that contribute to high von Neumann entropy.

These relationships are illustrated in a set of experiments below, in which different distributions of eigenvalues are generated that are used to build matrices corresponding to these eigenvalues and, subsequently, the von Neumann entropy is computed.

**Figure 1:**
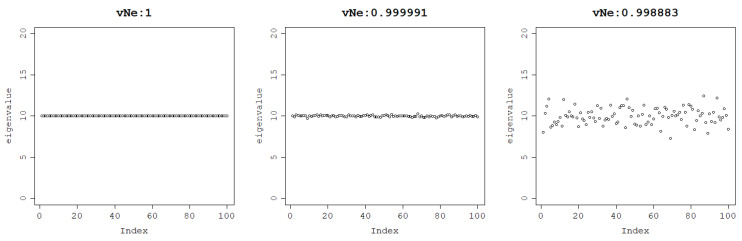
The von Neumann entropy of a matrix generated with a uniform eigenvalue distribution with no noise (**left**), Gaussian noise with standard deviation = 0.1 (**middle**), and Gaussian noise with standard deviation = 1 (**right**).

First, a set of 100 equal eigenvalues is generated. For a matrix built with such an eigenvalue distribution, the von Neumann entropy is 1 as the uncertainty of the system is maximum and the probabilistic interpretation of such a system is that the outcome of an experiment with such a probability distribution cannot be predicted. The von Neumann entropy remains very high when noise is added, which can be observed in Figure 1.

**Figure 2:**
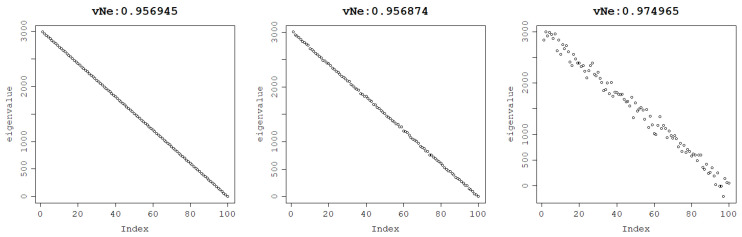
The von Neumann entropy of a matrix generated with eigenvalues decreasing linearly with no noise (**left**), Gaussian noise with standard deviation = 10 (**middle**), and Gaussian noise with standard deviation = 100 (**right**).

One could suggest that what makes the von Neumann entropy increase is the distribution of the eigenvalues and the relative differences between them and not their values themselves. However, when the distribution is unbalanced, say, when the eigenvalues decrease linearly, the von Neumann entropy remains very high both with and without noise, as depicted in Figure 2.

Since the linear distributions of the eigenvalues preserve significantly positive values (‘probabilities’), a more suitable function shape to test is a hyperbole. It is useful to look at von Neumann entropy of a matrix built with eigenvalues forming a $y=a+\frac{100}{x}$ hyperbole, where *a* is a vertical offset that can be modified.

**Figure 3:**
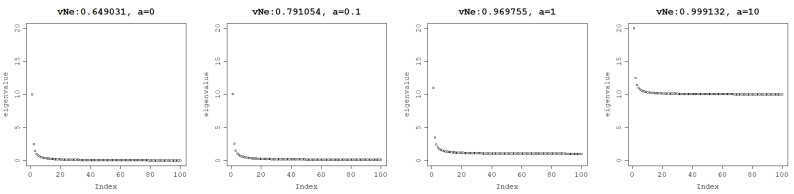
The von Neumann entropy of a matrix generated with eigenvalues decreasing along a hyperbole with varying offset ranging from a = 0, a = 0.1, a = 1 to a = 10, respectively from left to right.

When a=0, most of the eigenvalues are 0 and the von Neumann entropy is relatively low. Increasing *a* to 0.1 already slightly increases von Neumann entropy and this behavior is preserved when increasing the values of *a* to 1 and then to 10 when the maximum von Neumann entropy is reached. This proves that it is the magnitude of the eigenvalues, not their distribution that matters, as can be seen in Figure 3.

**Figure 4:**
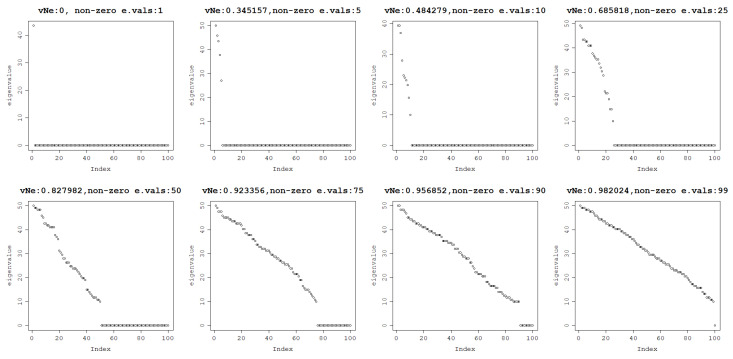
The von Neumann entropy of a matrix generated with a varying number of non-zero eigenvalues: 1, 5, 10, 25, 50, 75, 90, 99, respectively, from the top left to the bottom right.

As a final experiment, it can be investigated what proportion of non-zero eigenvalues is needed for high von Neumann entropy. When the number of non-zero eigenvalues is increased from 1 to 99 out of 100, the von Neumann entropy increases from 0 to almost 1. This relationship is illustrated in Figure 4.

It is clear that the more positive the eigenvalues, the higher the von Neumann entropy. Therefore, if the aim is to increase the von Neumann entropy of a matrix, an increase in the number of positive eigenvalues must be targeted.

### 2.3. Influence of Normalization and Data Scaling on Eigenvalue Distribution

Knowing what the desired properties of the kernel matrix eigenvalue distribution are, the influence of certain operations that change the kernel matrix can be examined in order to ensure obtaining its optimal characteristics.

Two operations explored are: kernel matrix normalization defined as in Equation (8) and scaling the rows of the data matrix (without the target variable) to unit length. As already established, the eigenvalues themselves are not of interest when targeting the high von Neumann entropy and the desired eigenvalue distribution has as many significantly positive eigenvalues as possible. Therefore, the preferred distribution of the kernel matrix eigenvalues is the least steep one, when the eigenvalues are sorted decreasingly.

It is found that both normalizing the kernel matrix as well as scaling the data rows to unit length have a positive effect on the eigenvalue distribution, as can be seen in the plots, meaning that they increase the number of non-zero eigenvalues. Normalizing the kernel matrix additionally scales the eigenvalues themselves to the same order of magnitude for all datasets, which is a desirable property.

For the RBF kernel with an arbitrarily chosen value of σ=1, the impact of row scaling is very small. Since the kernel matrix generated by the RBF kernel is by construction already normalized, its eigenvalues are of the same order of magnitude with and without data row scaling.

The results suggest that row scaling has a negative effect on the eigenvalue distribution, which matches the results for the polynomial kernel: both normalizing the kernel matrix as well as row scaling are beneficial for eigenvalue distribution for the polynomial kernel, but row scaling resulted in a less desirable distribution than kernel matrix normalization.

### 2.4. Influence of Normalization and Data Scaling on the von Neumann Entropy

It is worth noting that if a kernel function depends *continuously* on its hyperparameter, the von Neumann entropy of the kernel matrices it generates is a continuous function of the kernel hyperparameter too. (This is because the von Neumann entropy is determined exclusively by the kernel matrix eigenvalues and the eigenvalues are proven to be continuous functions of the kernel matrix entries, see [27]). In particular, since both the RBF kernel and the normalized polynomial kernel depend continuously on their hyperparameters and a composition of continuous functions is continuous, the von Neumann entropy is indeed a continuous function of σ and *d* respectively. This result allows us to model the von Neumann entropy in relation to kernel hyperparameters using a curve obtained from the data.

**Figure 5:**
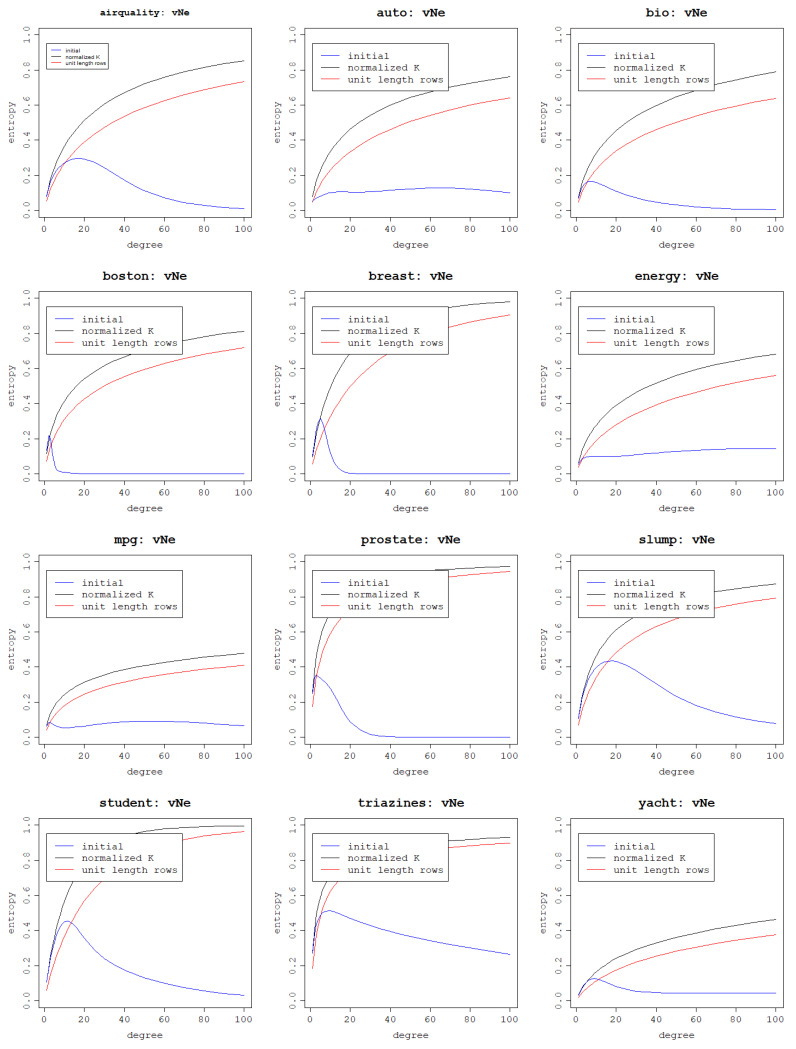
Effect of normalizing the kernel matrix and scaling data rows to unit length on the von Neumann entropy, depending on the degree of the normalized polynomial kernel per dataset.

**Figure 6:**
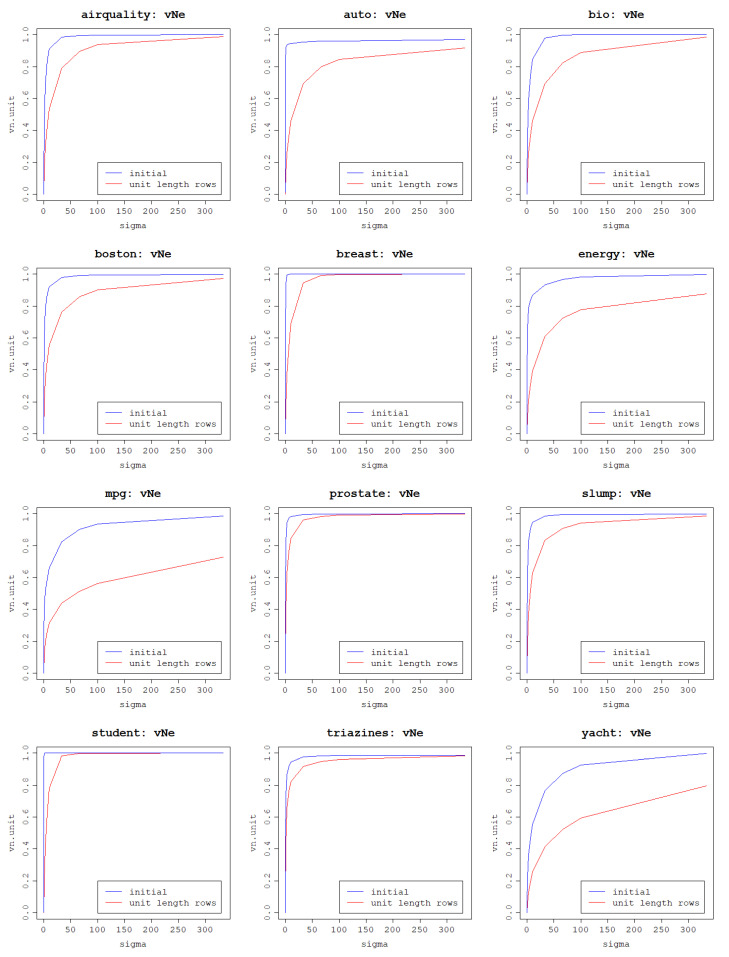
Effect of scaling data rows to unit length on the von Neumann entropy depending on the kernel width of the RBF kernel per dataset.

In this section, the impacts of kernel matrix normalization and row scaling are investigated on the relationship between the polynomial kernel degree and the von Neumann entropy. It is to be expected that these operations should increase von Neumann entropy as it has already been shown that they have a positive effect on the eigenvalue distribution. The results are presented in Figure 5 for the polynomial kernel and Figure 6 for the RBF kernel.

The plots for the polynomial kernel prove this hypothesis correct, but another very important result of both operations is strikingly visible: von Neumann entropy becomes monotonic and increases along with the kernel degree. Scaling rows to unit length yields slightly lower von Neumann entropies but the shape of the curve is very similar to the curve corresponding to normalized kernel matrix results. Since the RBF kernel matrix is already normalized, the benchmark eigenvalue distribution is missing and the comparison can only be made between the initial RBF kernel matrix eigenvalue distribution and the one with scaled rows. Thus, lower von Neumann entropy values are expected for the second case, which is confirmed by the plots.

It may seem that since unit scaling of the data rows has a negative impact on the eigenvalue distribution for the RBF kernel and a positive, but worse than the kernel matrix normalization, and the effect for the polynomial kernel, it is an undesired operation. However, one thing that becomes clear when examining the plots is that an increase in the von Neumann entropy as a result of the increase in the parameter value is more gradual and, thus, more control can be gained over the impact of kernel parameters on the von Neumann entropy. This means that if a specific range of the von Neumann entropy values is targeted, it can be more precisely found by testing different parameter values and the von Neumann entropy values will not change drastically with a small change in the parameter value. It is more desirable to be able to increase the kernel parameter values gradually and obtain a respective increase in the von Neumann entropy than obtain high entropies for small parameter values and not be able to fine-tune the parameter values to target the desired von Neumann entropy values. Furthermore, high values of the von Neumann entropy are still reached when scaling the data rows, but simply for higher kernel parameter values, which is what ultimately matters. This is not the case for the polynomial kernel in the initial setting when neither the kernel matrix was normalized nor were the data rows scaled.

For these reasons, both operations are accepted as resulting in a beneficial distribution of eigenvalues and yielding a desirable relationship between kernel parameters and the von Neumann entropy, and for further analysis, the rows of the data matrix are scaled to unit length and, subsequently, the kernel matrix is normalized. For the polynomial kernel, performing both of these transformations results in the same output as when just scaling the rows due to the kernel definition and division by 1 in the kernel normalization formula. For the RBF kernel normalization is ensured by the kernel itself but the rows are scaled beforehand.

**Figure 7:**
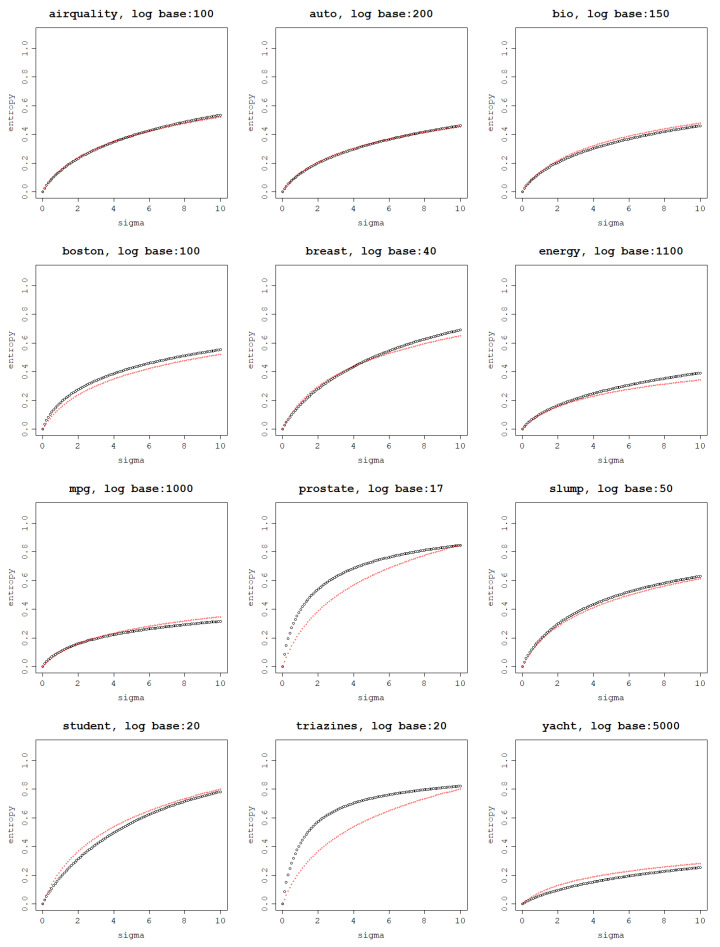
Modeling the relationship between the RBF kernel width and the von Neumann entropy (in black) using a logarithm of a chosen base (in red) per dataset.

An additional scenario when examining the von Neumann entropy as a function of the kernel parameter attempts to model it using a known function. The plots unanimously suggest this relationship can be estimated using a logarithm. To illustrate this, the von Neumann entropy is plotted against the value of the RBF kernel width σ with scaled rows for each of the analyzed datasets and 100 different σ parameter values between 0 and 10 in Figure 7. A logarithm of a chosen base is added to each plot that best describes the curve (in red).

**Figure 8:**
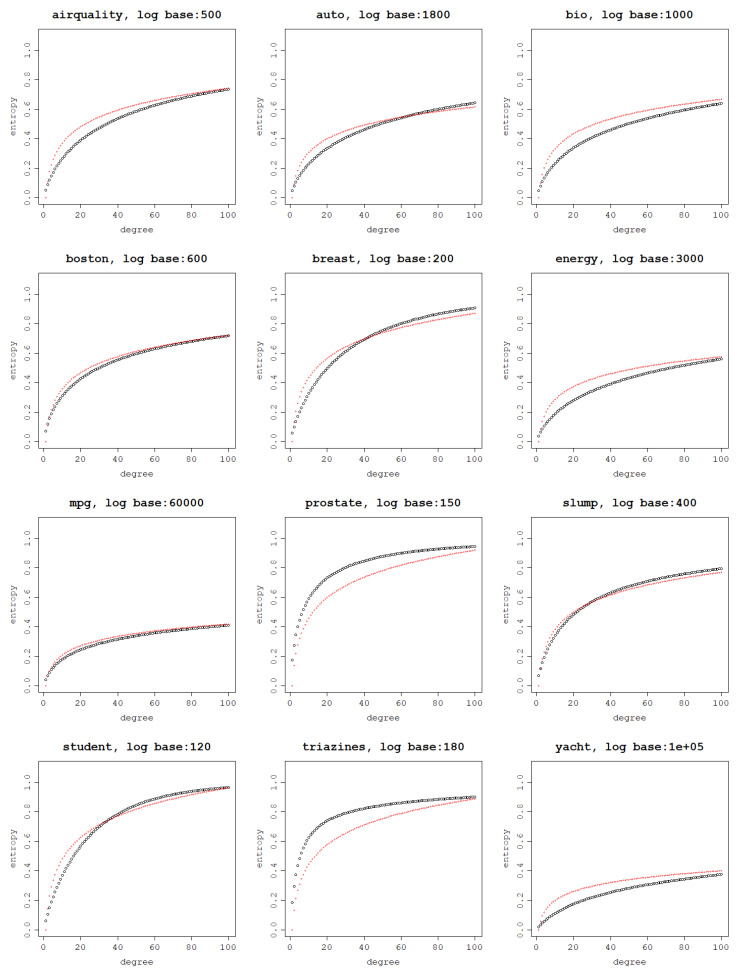
Modeling the relationship between a normalized polynomial kernel degree and the von Neumann entropy (in black) using a logarithm of a chosen base (in red) per dataset.

The same approximation can be done for the normalized polynomial kernel, again with data rows scaled to unit length and presented in Figure 8.

The logarithmic relationship between the von Neumann entropy of a kernel matrix and a kernel parameter is first depicted for exploration purposes. A link between the desired model properties (for instance the model’s fitting ability) and the von Neumann entropy of its corresponding kernel matrix can be extremely useful when working with kernel functions for which the properties of their hyperparameters are not known and their relationship with the fitting ability is not obvious. This way, costly cross-validation techniques can be avoided.

### 2.5. Influence of Normalization and Data Scaling on Condition Numbers

The notion of the condition number of a matrix is widely known and considered a measure of how well-defined a matrix is. It was first introduced by Alan Turing in [28] and further studied by von Neumann and Goldstine in [29]. It is defined as follows:

(14) $$\kappa \left(A\right)=∥A∥\cdot \;∥A^{-1}∥$$

where *A* is a real or complex matrix and ∥A∥ denotes the matrix norm defined as:

(15) $${∥A∥}_{d}=\underset{x\neq 0}{\sup}\frac{{∥Ax∥}_{d}}{{∥x∥}_{d}}$$

where ${∥\cdot ∥}_{d}$ is a vector d-norm (d≥1) and *x* is a vector belonging to the space where the vector norm is defined. For any matrix *A*, its condition number is κ(A)≥1 and for an identity matrix—−κ(I)=1. As described in [30], the condition number should be viewed as a value related to a matrix problem rather than a matrix alone. The authors describe that infinite values of the condition number indicate that the problem is *ill-posed*. For a matrix *A*, which is an instance of a problem *p*, infinite values of κ(A) mean that the matrix is singular and therefore the problem *p* of solving a linear system given by *A* is ill-posed. Hence, the higher the condition number, the closer the matrix is to being singular. Close-to-singular matrices are sources of serious numerical issues and should be generally avoided. Therefore, decreasing the condition number of a matrix is the desired effect. Computing the condition number for the kernel matrices used in all the performed experiments before and after normalizing the kernel matrix shows that indeed, the condition number decreases as a result of the normalization of the kernel matrix generated with the polynomial kernel.

As was the case with von Neumann entropy, scaling the data rows to unit length has a positive effect when compared to the non-scaled case, but yields worse results compared to the normalized kernel matrix case. Hence, when analyzing the results for the RBF kernel, row scaling results in higher condition number compared to the non-scaled (but, by construction, normalized) case.

## 3. Experimental Work

### 3.1. Methodology

Experiments are performed on multiple datasets in the *R* environment and RVM models are fit using the *kernlab* package. Some basic descriptor for the datasets are given in Table 2. The error metric for regression normalized mean square error (NMSE) is chosen, defined as $\frac{MSE}{Var(y)}$, where *y* is the target variable vector that the predictions are made on. The RBF and normalized polynomial kernel are used, respectively, with 24 parameter values of σ between 0.0001 and 6666 increasing in an approximately logarithmic manner, and 46 values of degree ranging from 1 to 2000 in an approximately logarithmic manner.

For every kernel parameter, each dataset is split 30 times into a training set used to build the model and a test set on which the model’s performance is evaluated. The train–test splits are done in proportions 75/25. Therefore, for each value of the kernel parameter 30 values of training NMSE and test NMSE are obtained along with the value of the von Neumann entropy of the kernel matrix corresponding to the parameter.

No cross-validation is needed since RVM is used instead of SVM, which eliminates the influence of additional parameters ϵ and *C* that would be present if using an SVM. Prior to modeling, all datasets were slightly preprocessed by encoding categorical variables and imputing or removing missing values when needed. Both numerical input variables and target variables were scaled using their standard deviations, but not centered by their means.

### 3.2. Datasets

The following problems (given by the available datasets) are used in the experiments:

- New York air quality measurements, denoted as **air quality**: daily air quality measurements in New York, May to September 1973
  - Response variable: ozone
  - Source: UC Irvine Machine Learning Repository
- Automobile dataset, denoted as **auto**: 1985 auto imports database
  - Response variable: price
  - Source: UC Irvine Machine Learning Repository
- Biosensor dataset, denoted as **bio**: electrochemical readings from second-generation glucose oxidase amperometric biosensor
  - Response variable: biosensor output
  - Source: https://link.springer.com/chapter/10.1007/978-3-319-13650-9_40 (accessed on 10 June 2020)
- Boston Housing Data, denoted as **Boston**: housing data for 506 census tracts of Boston from the 1970 census
  - Response variable: medv
  - Source: R package *MASS*
- Breast Cancer Wisconsin (Prognostic) dataset, denoted as **breast**: follow-up data for breast cancer cases seen in patients by Dr. Wolberg since 1984
  - Response variable: time
  - Source: UC Irvine Machine Learning Repository
- Energy efficiency dataset, denoted as **energy**: energy analysis data using 12 different building shapes simulated in Ecotect
  - Response variable: heating load
  - Source: UC Irvine Machine Learning Repository
- Auto MPG dataset, denoted as **mpg**: data concerning city-cycle fuel consumption in miles per gallon
  - Response variable: mpg
  - Source: UC Irvine Machine Learning Repository
- Prostate Cancer Data, denoted as **prostate**: data related to a study that examined the correlation between the level of prostate-specific antigen and a number of clinical measures in men who were about to receive a radical prostatectomy.
  - Response variable: lpsa
  - Source: R package *lasso2*
- Concrete slump test dataset, denoted as **slump**: the concrete slump flow data with measurements of water content and other concrete ingredients
  - Response variable: 28-day compressive strength
  - Source: UC Irvine Machine Learning Repository
- Student performance dataset, denoted as **student**: data concerning student achievement in secondary education at two Portuguese schools
  - Response variable: final grade (G3)
  - Source: UC Irvine Machine Learning Repository
- Triazine data, denoted as **triazines**: pyrimidine QSAR dataset containing created to predict the inhibition of dihydrofolate reductase by pyrimidines based on quantitative structure–activity relationships
  - Response variable: activity
  - Source: https://www.dcc.fc.up.pt/~ltorgo/Regression/DataSets.html (accessed on 20 June 2022)
- Yacht hydrodynamics dataset, denoted as **yacht**: Delft dataset, used to predict the hydrodynamic performance of sailing yachts from dimensions and velocity
  - Response variable: residuary resistance
  - Source: UC Irvine Machine Learning Repository

### 3.3. Relation between the von Neumann Entropy and Model Fitting Ability

In a series of experiments conducted using the normalized polynomial kernel and RBF kernel it is shown how an increase in the von Neumann entropy results in a decrease in the training mean square error. This behavior is present for all 12 analyzed datasets when fitting an RVM model with a set of kernel parameters and performing a train–test split 30 times for each parameter value.

**Figure 9:**
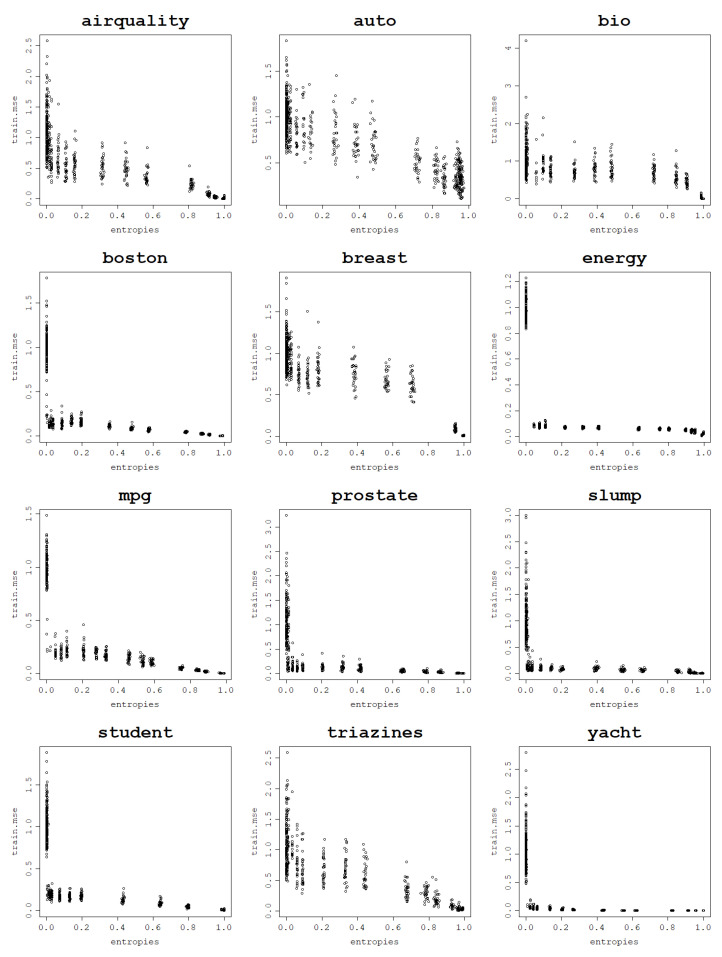
Fitting ability of an RVM model with the RBF kernel: training NMSE against the von Neumann entropy across 30 train–test split folds.

**Figure 10:**
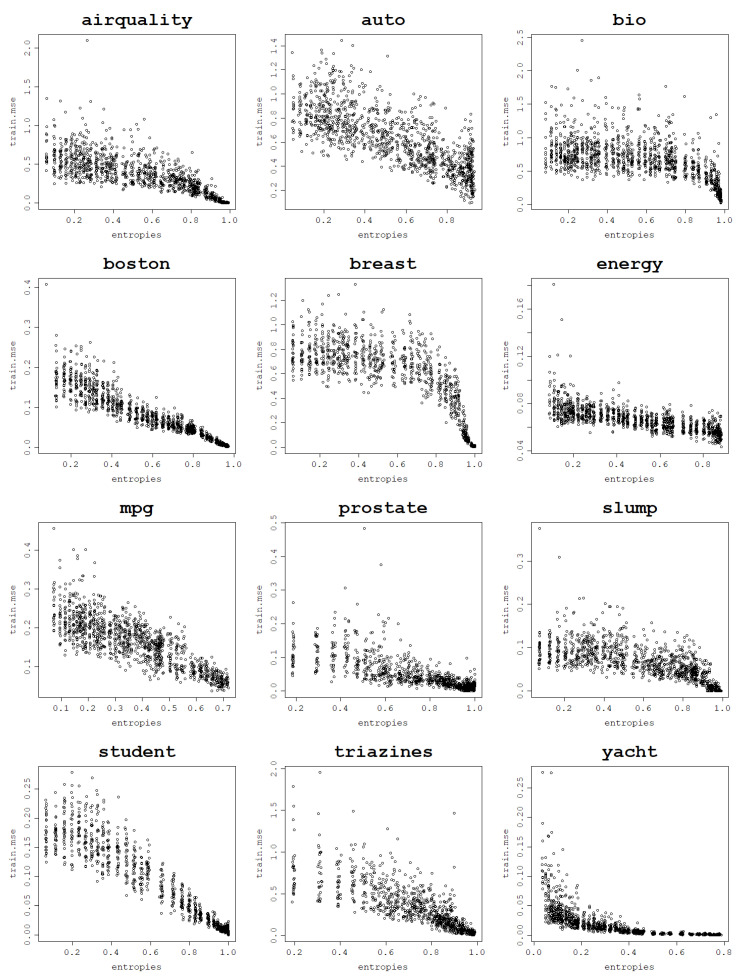
Fitting ability of an RVM model with the normalized polynomial kernel: training NMSE against the von Neumann entropy across 30 train–test split folds.

Plots per dataset of the training NMSE against the von Neumann entropy over 30 train–test splits for each kernel parameter value are presented first for the RBF kernel in Figure 9. The same relationship exists for the normalized polynomial kernel, which can be seen in Figure 10.

Computing correlations between the von Neumann entropy and training NMSE values proves a strong negative link between them across all datasets. Both Pearson and Spearman correlations are computed in order to account for all sorts of monotonic relationships, not just the linear ones, and the results are presented in Table 3 for the RBF kernel and in Table 4 for the normalized polynomial kernel. The plots, as well as the correlation coefficients, prove that a strong dependence can be established between the von Neumann entropy and the training NMSE.

For most datasets, von Neumann entropy values lower than 0.2, and often close to zero, are reached for very low σ and degree values. For these parameter values, the von Neumann entropy is the lowest and the training NMSE is the highest among all von Neumann entropy values, which suggests that these models are ’underfitting’. For the RBF kernel training, NMSE values corresponding to the von Neumann entropy close to zero are so high that the disproportions with the training NMSE for moderate and high von Neumann entropy values become huge.

With an increase in the von Neumann entropy, training NMSE decreases continuously, usually in an approximately linear manner, until it reaches values so low that they are effectively zero for very high von Neumann entropy values. For the normalized polynomial kernel and some datasets such extreme training NMSE values are already reached for von Neumann entropy values around 0.7–0.8. When the von Neumann entropy approaches 1, training NMSE approaches 0, and for such high values of the von Neumann entropy, the corresponding models are highly ’overfitting’, according to the previously established relationship between the von Neumann entropy and matrix properties.

Highly overfit models form kernel matrices that are close to an identity matrix that has the von Neumann entropy close to 1. Since the polynomial degree can be arbitrarily increased up to the data size (and beyond though with no further increase in the von Neumann entropy) and the σ value can be arbitrarily large, these extremely large values of the von Neumann entropy can be reached for each dataset. This reflects the cases when the data fit perfectly and are essentially memorized by the model, which is not the desired behavior.

#### Application to the ELM Kernel

RBF and polynomial kernels are the best known and widely used and therefore their properties and behavior have been thoroughly studied. As already mentioned, the relationship between the RBF kernel width σ and polynomial degree *d* and the fitting ability of models using these kernels is known. Therefore, the practitioner has an intuition about what parameter values are likely to result in under- or overfitting models. However, in practice, more specialized or more sophisticated kernel functions may be used depending on the analyzed data type and desired properties. In fact, the incredible flexibility and adaptability are the biggest advantages of kernel methods since they are not limited by the model input requirements and can operate on virtually any datum provided that a valid kernel function is designed and used.

Therefore, there is a need to generalize the understanding of the overfitting and underfitting propensities of the kernel models depending on their hyperparameters, beyond the most popular kernel functions. This way underfitting and overfitting can be avoided even when working with custom kernel functions. For that reason, linking the fitting ability of the model to the von Neumann entropy of the kernel matrix can be a useful indication of the model’s suitability for a given dataset. As an example, if the von Neumann entropy remains very low for all hyperparameter values, it can be deduced that the model is unable to fit the data.

**Figure 11:**
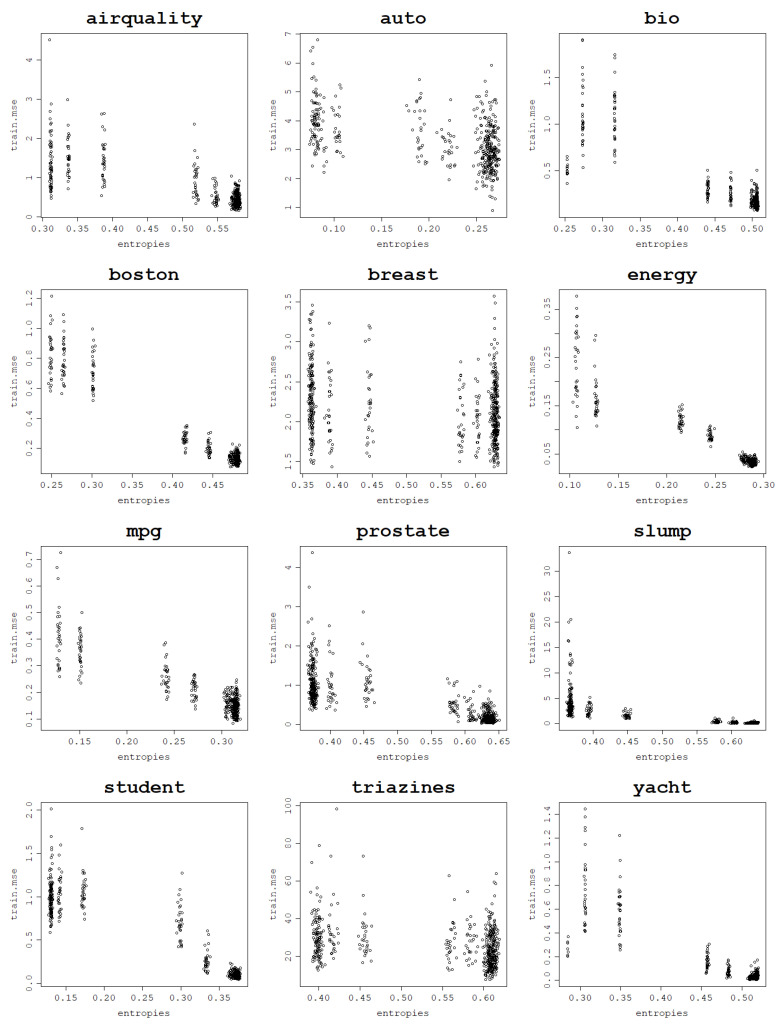
Fitting ability of an RVM model with an ELM kernel: training NMSE against the von Neumann entropy across 30 train–test split folds.

To illustrate that an increase in the von Neumann entropy is linked to a decrease in the training NMSE and, thus, an increase in the fitting ability of the RVM, the behavior of the asymptotic ELM kernel described in Equation (1) is analyzed for the 12 datasets. Plots of the training NMSE against the von Neumann entropy are presented in Figure 11 and the correlations can be found in Table 5.

### 3.4. Relation between the von Neumann Entropy and Model Generalization Power

After establishing the link between the model’s fitting ability and the von Neumann entropy of the corresponding kernel matrix it becomes clear that increasing the von Neumann entropy results in a more flexible model that can basically memorize the dataset. This is a useful property since it lets the modeler discard models that have no fitting power and will underfit the data.

However, the more painful issue is usually the risk of overfitting, not underfitting. Knowing how the von Neumann entropy relates to training error, models with high von Neumann entropy kernel matrices can be expected to highly overfit the data since they aim to minimize the training error. Therefore, it is easy to predict that kernel matrices with very high von Neumann entropies will not generate models that generalize well and that the optimal von Neumann entropy values that generate models with the lowest test errors and, thus, the highest prediction power should lie in the moderate von Neumann entropy value range.

**Figure 12:**
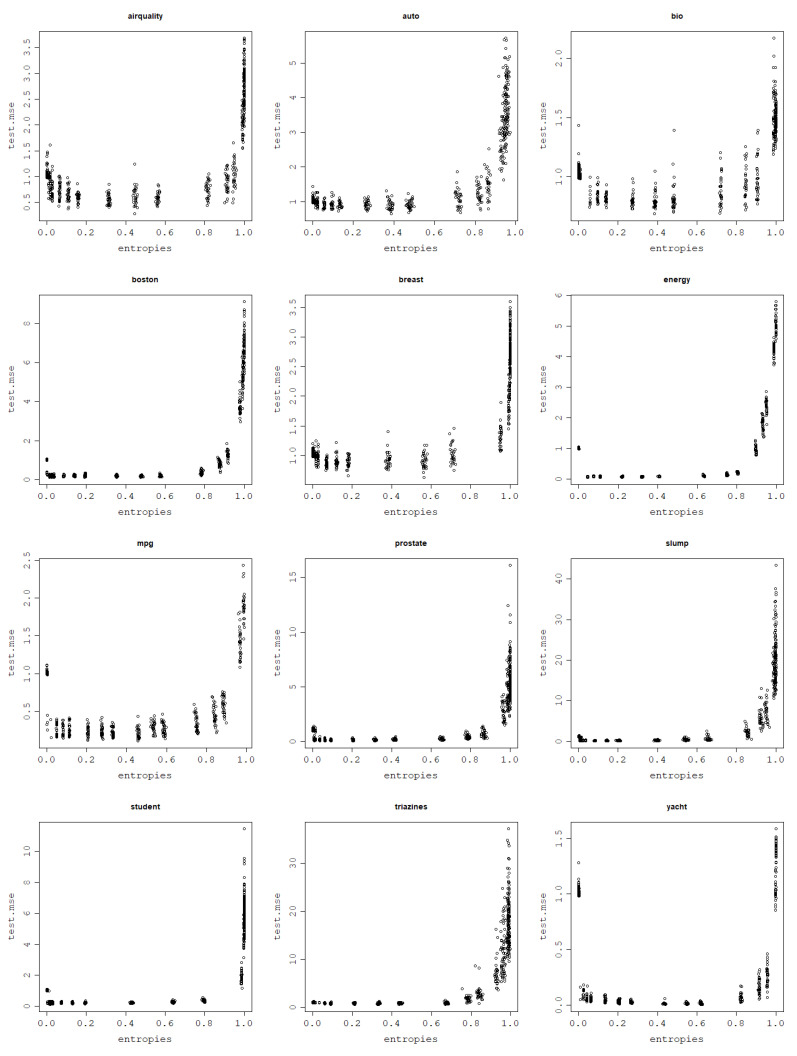
Generalization power of an RVM model with the RBF kernel across 30 train–test split folds.

**Figure 13:**
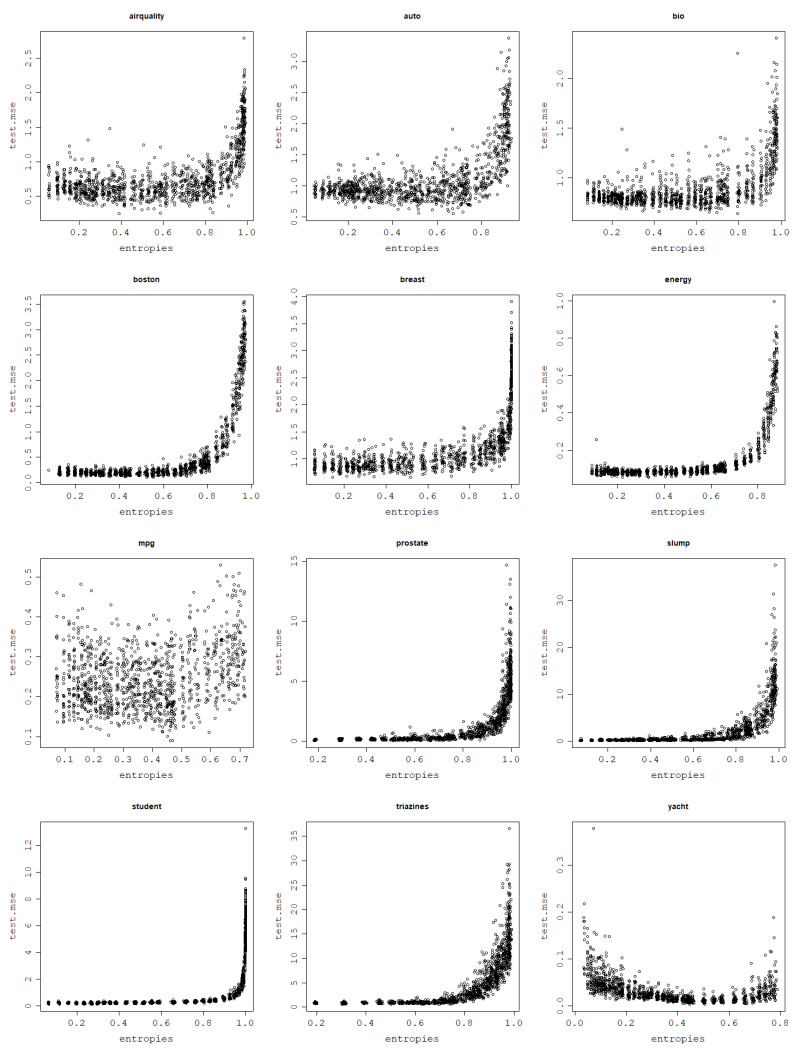
Generalization power of an RVM model with the normalized polynomial kernel across 30 train–test split folds.

Since low von Neumann entropy values correspond to underfit models, high ones correspond to overfit models, and the optimal ones are somewhere in between; a logical assumption to make is that the curve of the test mean square error against the von Neumann entropy should have a parabola-like shape. To confirm this initial intuition, plots of the test NMSE against the von Neumann entropy for the datasets are presented in Figure 12 for the RBF kernel and Figure 13 for the normalized polynomial kernel. Indeed, the plots confirm that high von Neumann entropy values are linked to high test NMSE, which, keeping in mind the results related to model fitting ability, clearly indicates overfitting. The lowest test NMSE is in turn achieved for moderate von Neumann entropy values and the transition between low and high von Neumann entropy values is relatively smooth.

**Figure 14:**
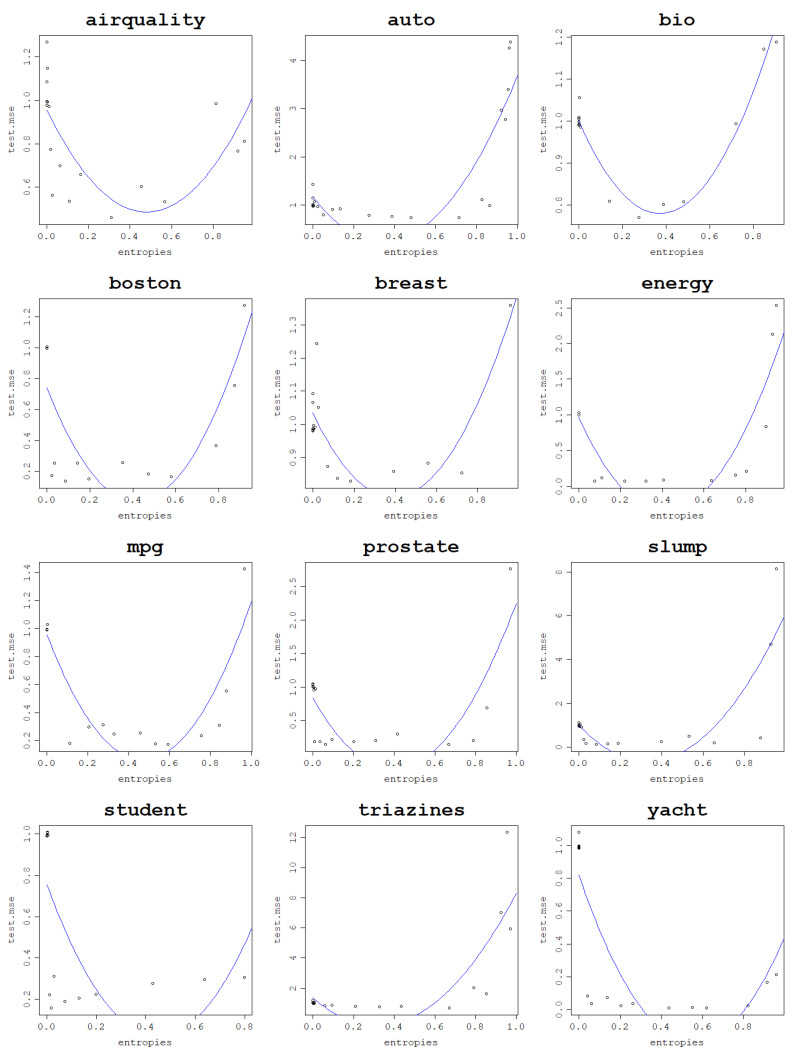
Generalization power of an RVM model with the RBF kernel on a single train–test split fold and a polynomial regression of the degree 2 curve.

**Figure 15:**
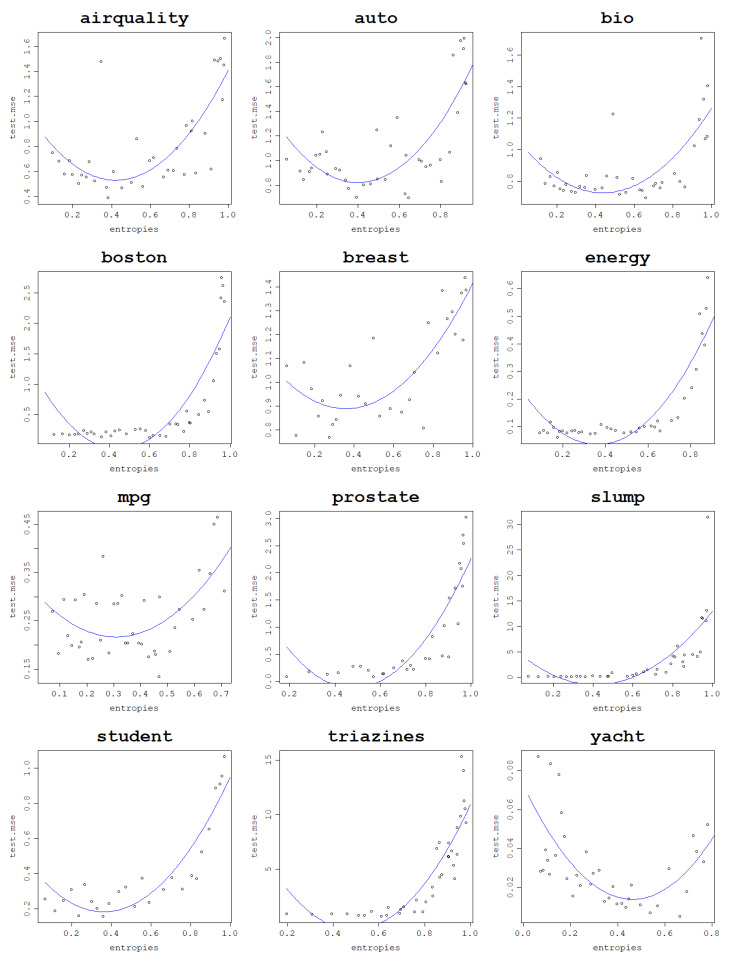
Generalization power of an RVM model with the normalized polynomial kernel a on a single train–test split fold and a polynomial regression of the degree 2 curve.

As a confirmation of this hypothesis, an arbitrary train–test split fold is chosen for each dataset for both models, and the test NMSE obtained in this fold is plotted against the von Neumann entropy of the kernel matrix built in this fold. Subsequently, a polynomial regression model of degree 2 is fit to the obtained data points and plotted as a curve to validate that the relationship is truly parabola-like. These plots can be seen in Figure 14 for the RBF kernel and Figure 15 for the normalized polynomial kernel.

**Figure 16:**
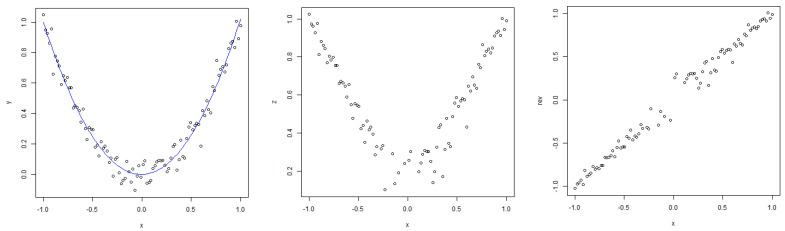
Illustration of the procedure used to transform a non-monotonic relationship into a monotonic one.

For the polynomial kernel, the curvature is only one-sided for some datasets since the lowest degree tested is d=1 and it may not lead to underfitting, such as low values of σ for the RBF kernel that can have its value decreased to an arbitrarily low positive number. Although the graphs suggest a clear relationship that holds across all datasets, measuring a quadratic relationship analytically is not as straightforward as in the previous, case since the relationship is generally not monotonic; therefore, simple correlations are not able to pick up the patterns present in the data. One idea on how to measure the strength of this link is by *making* these relationships monotonic to be able to use correlation coefficients. Since the curve appears to be parabola-like, the transformation used is the following: fit a polynomial regression model of degree 2 to the existing test NMSE against the von Neumann entropy data, find the quasi-parabola’s breakpoint, i.e., the minimum of the estimated degree 2 model, take the square root of the data and, subsequently, reverse the sign of the test NMSE values for von Neumann entropy values lower than the estimated minimum. An example of this procedure is shown in Figure 16.

Following this procedure, the relationship between the von Neumann entropy and test NMSE can be made monotonic and measured using standard correlation coefficients. The resulting plots are disrupted at the ‘breakpoint’ due to the fact that test errors close to 0 are generally not obtained.

**Figure 17:**
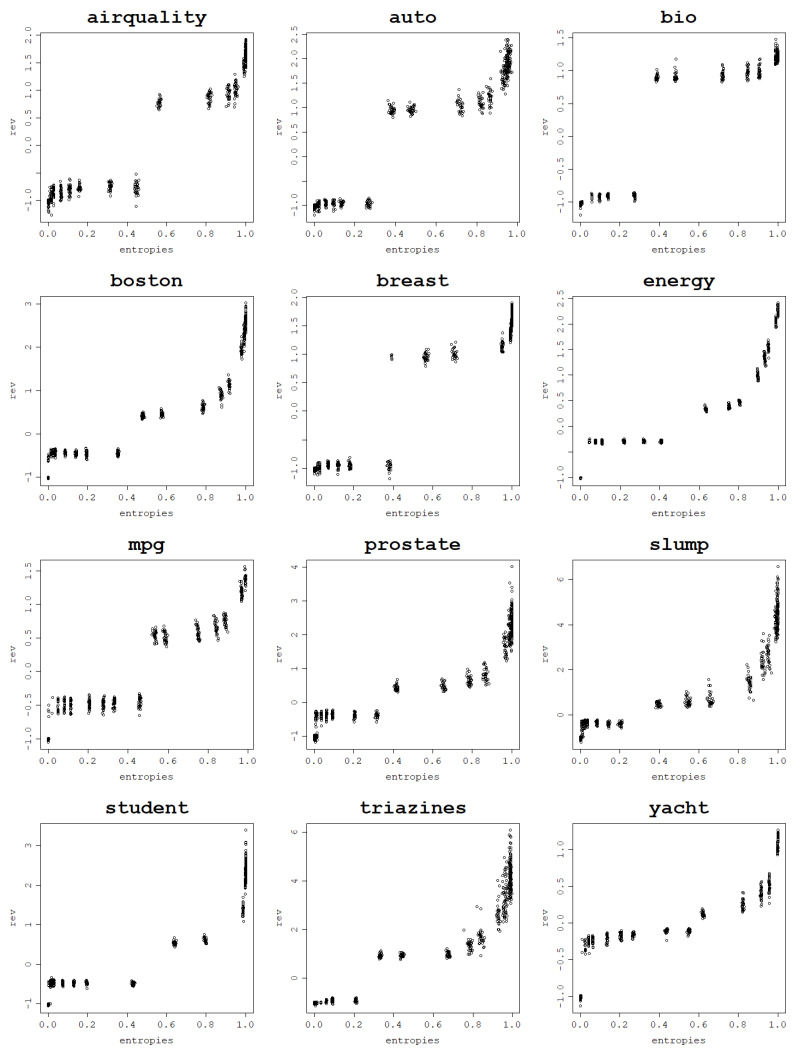
Relationship between the von Neumann entropy and test NMSE for the RBF kernel after taking a square root and reversing the NMSE sign to the left of the minimum of the parabola.

**Figure 18:**
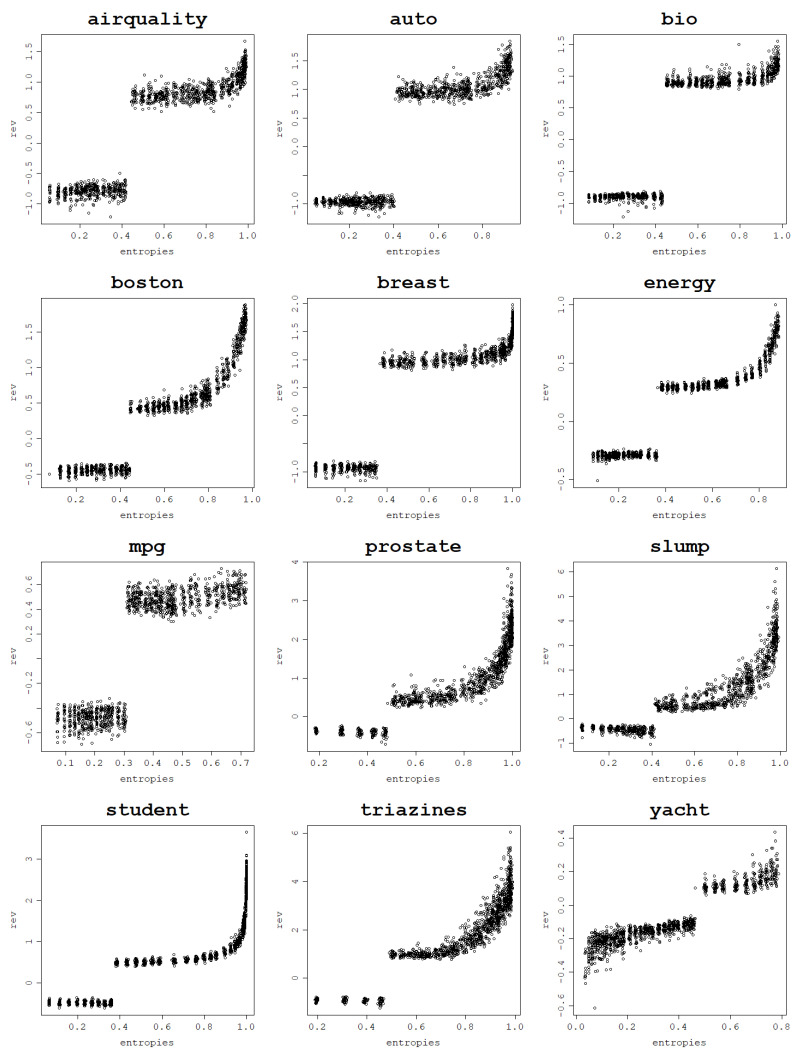
Relationship between the von Neumann entropy and test NMSE for the normalized polynomial kernel after taking a square root and reversing the NMSE sign to the left of the minimum of the parabola.

Moreover, for the polynomial kernel, low-degree values do not tend to underfit the data as much as in the case when using the RBF kernel with very low σ values and, therefore, the plots show a flat pattern for low degrees rather than a curved one. It is clear though that increasing the degree leads to overfitting and high test errors. The monotonic results of the described procedure can be seen in Figure 17 for the RBF kernel and in Figure 18 for the normalized polynomial kernel.

Spearman and Pearson correlation coefficients are computed as measures of what used to be curvatures visible in the plots. The correlations appear to be very strongly positive, suggesting that before taking the square root and reversing the sign at the test NMSE minimum, the relationship could actually be described as quadratic. The correlation scores are presented in Table 6 for the RBF kernel and in Table 7 for the normalized polynomial kernel.

### 3.5. Entropy Range for Optimal generalization Performance

Providing a heuristic for the optimal von Neumann entropy value independently of the dataset would vastly decrease the cost of the parameter search and would mean that a practitioner only needs to aim at these particular von Neumann entropy values when building a kernel model. It appears that such a range of close-to-optimal von Neumann entropy values can indeed be found across the analyzed datasets for both kernel functions tested.

The heuristic for the optimal von Neumann entropy value is defined as the minimum of the polynomial model of degree 2 fit the data consisting of the von Neumann entropy and test NMSE values. Entropy values corresponding to the minimum appear to always be between 0.3 and 0.5, with one exception when the value is 0.55. These minimum arguments of the previously fit quadratic function, i.e., the heuristic for the optimal von Neumann entropy values are presented in Table 8 and Table 9.

### 3.6. Golden-Section Search for Optimal von Neumann Entropy Values

As previously shown, the relationship between the von Neumann entropy and the kernel width σ for the RBF kernel and degree *d* for the normalized polynomial kernel is monotonic and an increase in the parameter value results in an increase in the von Neumann entropy.

Since it has been already established that von Neumann entropies between 0.3 and 0.5 should be targeted for close-to-optimal generalization performances of RVM models, the missing piece is to find a parameter value that generates a kernel matrix with the von Neumann entropy value in this range.

The relationship between the von Neumann entropy of a kernel matrix and a kernel parameter appears to follow a logarithmic curve as previously shown; therefore, one idea would be to try to model the relationship using a logarithm by estimating the logarithm base appropriate for a given dataset. In a mathematical sense, to find the base of a logarithm, only one point is needed; logarithms always take the value of 0 for the argument value 1 and the base can be found by calculating:

(16) $$\log_{b}x=y\Leftrightarrow b^{y}=x\Leftrightarrow b=\sqrt[{y}]{x}$$

Since von Neumann entropy values are positive, only base values b≥1 can be considered. However, since the data does not represent this ideal case, one should certainly not rely on a single kernel parameter value to model the relationship with von Neumann entropy. To fit the logarithmic model a modeler would have to cautiously choose a few parameter values, such that at least one of them generates a kernel matrix with von Neumann entropy lower than 0.3, and at least one of them generates a kernel matrix with von Neumann entropy higher than 0.5. Since von Neumann entropy is a continuous function of the kernel parameters, a parameter that yields von Neumann entropy within the optimal range of 0.3–0.5 will lie in between these two parameters.

Although this approach looks promising, it appears that the number of points needed to actually estimate the logarithm would be too high to be a viable alternative to a regular grid search. This is due to the fact that the logarithmic nature of the link between the von Neumann entropy and kernel parameters is (in most cases) displayed in a certain range, either for small parameter values or for large ones. However, the goal is not to perform an expensive search through dozens of hyperparameters, since this would entail computing the von Neumann entropy by finding the kernel matrix eigenvalues.

A better idea for a way of finding a value inside the desired von Neumann entropy range is leveraging the quadratic nature of the relationship between test NMSE and the von Neumann entropy and applying a modification of the *golden-section search* technique. The golden-section search is used to find the extremum of a strictly unimodal function in a series of iterations by narrowing the range containing the extremum at each iteration [31,32]. At every step, three points that form a golden ratio $\varphi =\frac{1+\sqrt{5}}{2}$ are defined and used to determine the range where the extremum exists.

Since the objective of this section is finding a value within a given range, not an extremum, the algorithm is slightly modified to suit the purpose, but the basic idea remains the same. A detailed description of this approach is presented below.

1. Choose the initial range of kernel parameters [a,b] to search through.
2. Compute vNe(a) and vNe(b), and if one of them amounts to a value between 0.3 and 0.5, stop. The corresponding parameter value is the sought approximation for the optimal value.
3. Compute $c=b-\frac{b-a}{\phi }$ and evaluate vNe(c). If vNe(c) amounts to a value between 0.3 and 0.5, stop; *c* is the sought approximation for the optimal value.
4. Compute $d=a+\frac{b-a}{\varphi }$ and evaluate vNe(d). If vNe(d) amounts to a value between 0.3 and 0.5, stop; *d* is the sought approximation for the optimal value.
5. If:
  - vNe(c) <0.3, vNe(d) >0.5 and |vNe(c) − 0.3|< | vNe(d) − 0.5| or
  - both vNe(c) >0.5, vNe(d) >0.5
  that is, if vNe(c) is closer to the optimal range than vNe(d), shift the points defining the search interval by substituting b=d
6. Otherwise, if:
  - vNe(c) <0.3, vNe(d) >0.5 and |vNe(c) − 0.3|≥ | vNe(d) − 0.5| or
  - both vNe(c) <0.3, vNe(d) <0.3
  that is, if vNe(d) is closer to the optimal range than vNe(c), shift the points to be a=c
7. Go back to point 3

Taking this into account, managing to find a good enough logarithmic fit is no longer necessary, but the number of parameters tested will depend on the computational resources available, dataset size, and optimality requirements. In some cases, one will aim at targeting the von Neumann entropy values as close as possible to the middle of the range and avoiding reaching the border von Neumann entropy values, other times just obtaining parameter values of the right order of magnitude will be enough. This idea is explored in subsequent experiments.

Following the steps defined before, initial values of the σ parameter for the RBF kernel are chosen to be a=1,b=33 as relatively moderate values that have a high chance to initially hit the right von Neumann entropy values. Very low σ values are likely to yield very low von Neumann entropy values as can be seen in previous results. The opposite is true for very high σ values. Similarly, the initial values of the degree for the normalized polynomial kernel are chosen to be a=1,b=70 for the same reasons. For these values, the von Neumann entropy of the corresponding kernel matrices for each dataset is computed.

Test NMSE values are obtained for the heuristic values of the parameters and are reported along with the number of points evaluated in the process and can be seen in Table 10 for the RBF kernel and Table 11 for the normalized polynomial kernel. The von Neumann entropies and test NMSE were computed using a 30-time train–test split and averaged. The true optimal parameter values are calculated as the ones obtaining the minimum average test NMSE over the 30 folds in the experiments from Section 3.3 and are presented alongside the heuristic results for comparison.

The heuristic results are very close to the optimal ones for most datasets. Both the RBF kernel and the normalized polynomial kernel struggled with the *slump* dataset, but for the rest of the datasets, the decrease in performance is very slight. Even when the heuristic kernel parameters are not close to the optimal ones, this disproportion is not reflected in the model quality and suggests that the range of kernel parameters that yield close-to-optimal performance is not very narrow.

In most cases, the von Neumann entropy for only a few kernel parameters had to be evaluated before reaching an acceptable value. Both for the RBF kernel as well as for the normalized polynomial kernel it was on average 5 parameters. Compared to the grid search performed when targeting optimal values, this number is lower by an order of magnitude with relatively little drop in the learning ability of the model.

Most importantly, these results were obtained within *minutes* for all datasets and, thus, required a fraction of the computational effort needed to obtain the exact optimum values. This is an enormous benefit given the low loss of quality: the loss in test error is 2.9% on average across all datasets while the cost of applying the heuristic for parameter tuning (without considering the RVM fit) is 4–5 times lower than the cost of the benchmark method. These findings allow us to postulate that the proposed heuristic approach is in fact an efficient and viable alternative to the exhaustive methods.

### 3.7. Application to a Middle-Sized Dataset: Abalone

The *Abalone dataset* was created to predict the age of the abalone from its physical measurements. The age of the abalone is determined by cutting the shell through the cone, staining it, and counting the number of rings through a microscope. The number of rings is the response variable and the problem can be treated as a classification or regression task.

The dataset is significantly larger than the previously analyzed datasets as it contains 4177 instances and 9 attributes making middle-sized. Training an RVM with kernel parameter-tuning on a dataset this size would take days of computational power and would be highly impractical. Therefore, the proposed heuristic for the choice of kernel parameters for optimal generalization performance of the RVM model becomes a real game-changer.

To prove that what we have presented so far holds for bigger datasets too, the same methodology is used to establish the links between kernel matrix von Neumann entropy and the model’s fitting ability and generalization power, but a 5-time train–test split is used rather than a 30-time train–test split due to very long modeling times.

**Figure 19:**
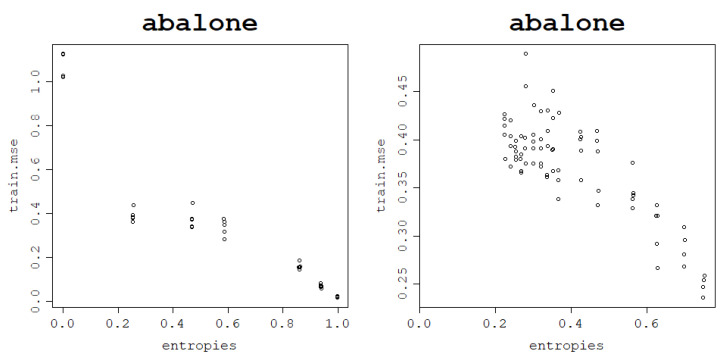
The fitting ability of an RVM model with the RBF kernel (**left**) and normalized polynomial kernel (**right**): training NMSE against the von Neumann entropy across 5 train–test split folds.

The results confirm that the behavior remains the same when increasing the data size as can be seen in Figure 19 for both kernels.

The correlations between the von Neumann entropy and training NMSE for both kernels are very strong and negative and can be inspected in Table 12.

**Figure 20:**
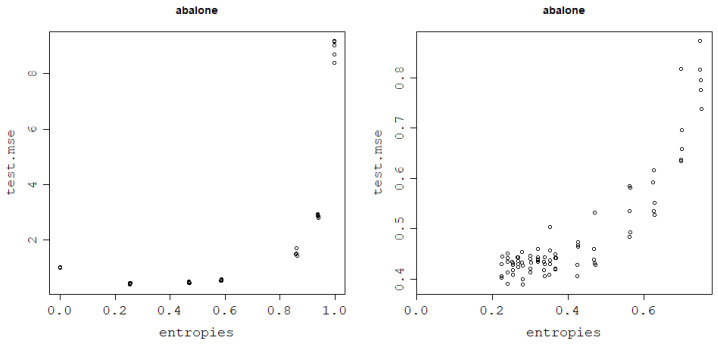
Generalization power of an RVM model with the RBF kernel (**left**) and normalized polynomial kernel (**right**): test NMSE against the von Neumann entropy across 5 train–test split folds.

Furthermore, as for the other datasets, the generalization power of the model depending on the von Neumann entropy of the kernel matrix follows a quadratic pattern, forming a curve that can be modeled using a polynomial of degree 2. Optimal values of the von Neumann entropy, i.e., the ones that guarantee the lowest test NMSEs, are moderate, with very high values leading to data overfitting for both kernel functions. These results can be seen in Figure 20 across all 5 train–test folds.

**Figure 21:**
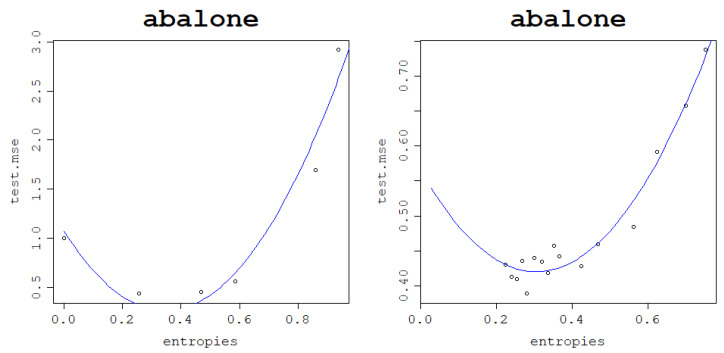
Generalization power of an RVM model with the RBF kernel (**left**) and normalized polynomial kernel (**right**) on a single train–test split fold and a polynomial regression of the degree 2 curve.

**Figure 22:**
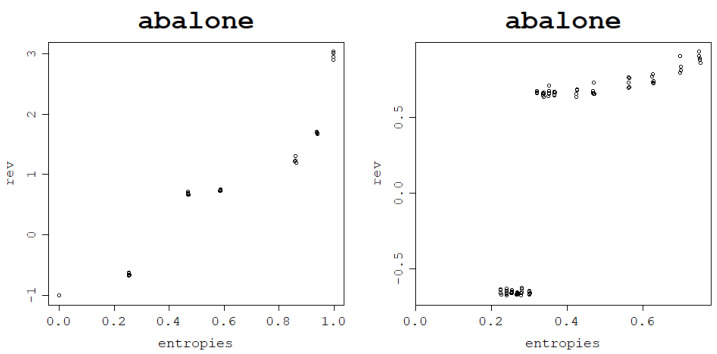
Correlation between the von Neumann entropy and test NMSE for the RBF kernel (**left**) and normalized polynomial kernel (**right**) after taking a square root and reversing the NMSE sign to the left of the minimum of the parabola.

Similar to before, the link between the von Neumann entropy and test NMSE can be modeled using a degree 2 polynomial regression model, as can be seen in Figure 21, and measured with correlation coefficients. After finding the minimum of the curve, taking a square root of the data points and reversing the sign of test NMSE for von Neumann entropy values lower than the minimum of the function, a pattern shown in Figure 22 is obtained. The Spearman and Pearson correlations for the resulting data are strongly positive and can be inspected in Table 13.

The minima of the parabolas fit in Figure 20 appear to be 0.33 for the normalized polynomial kernel and 0.36 for the RBF kernel. Thus, they fall into the range of 0.3–0.5, previously indicated as the heuristic for the optimal von Neumann entropy choice and, thus, support the applicability of the heuristic.

Now that it is shown that the findings of this work apply to bigger datasets, the same way as they do for small datasets, the presented heuristic can be applied to find the RBF σ value and polynomial degree value that generate the optimal model approximation. The same procedure as previously presented must be followed. The values of a=1,b=70 are chosen as initial parameter samples for the degree of the normalized polynomial kernel and it appears that for *b*, i.e., for degree=70, the corresponding von Neumann entropy value is 0.32 which lies inside the desired optimal von Neumann entropy range and the process finishes on the first iteration.

For the RBF kernel, the same σ values as before are tested: a=1,b=33. The value of the von Neumann entropy corresponding to *b* appears to be 0.406, which, again, lies inside of the optimal von Neumann entropy range and the procedure stops. Table 14 and Table 15 show how these results compare to the optimal results found in an exhaustive search.

Performance obtained using the proposed heuristic is slightly worse but comparable with the optimal performance. Although the values of σ suggested by the heuristic are three times as high as the optimal ones, it appears that the difference in NMSE is small. The same applies to the normalized polynomial kernel. The degrees are quite different but the test NMSE values are close to optimal.

In this particular case, the golden-section search only required evaluating two data points, the initial values, due to the fact that the dataset required complex models and thus high degrees and σ values. For another dataset, more values would possibly have to be tested but as shown in the experiments on the small datasets this number tends to be—on average—5. This means that instead of scanning through a grid of 16 σ values and 32 degrees, one can evaluate the von Neumann entropy on a few wisely chosen parameter samples and obtain close-to-optimal results. By doing so, the modeler vastly reduces the time needed to find a good model with little loss in model quality.

## 4. Conclusions and Future Work

The relationship between the von Neumann entropy of a kernel matrix and the generalization power of an RVM was studied. A link was sought between the von Neumann entropy and test MSE and, thus, the learning ability of the model. A range of good entropy values is demonstrated that leads to close-to-optimal generalization results, as compared with those found by standard methods.

As shown in the experimental part of the paper, the von Neumann entropy is strongly related to RVM model flexibility. Low von Neumann entropy values of the kernel matrix indicate a simple, possibly underfit model and are generally obtained for relatively low σ values for the RBF kernel and degrees for the normalized polynomial kernel. On the other hand, high von Neumann entropy values—especially those close to 1—are reached for high kernel parameter values and indicate a very flexible, possibly overfit model. None of these extremes is good for the model generalization power and learning ability. However, values of the von Neumann entropy in the range of 0.3 to 0.5 tend to be obtained for close-to-optimal models in terms of the generalization ability and thus this interval should be targeted when building the kernel matrix. Finding kernel parameters that yield von Neumann entropy values in this range is not trivial, but can be achieved by employing a modified version of the golden-section search algorithm. This way, close-to-optimal test NMSE values can be obtained, even though the computational effort is reduced several times.

The proposed heuristic makes it possible to overcome the arguably largest limitation of the kernel methods: the long time needed to fine-tune the kernel hyperparameters that greatly influence the model’s performance. Gaining efficiency in this area is, therefore, an important step toward making kernel-based machine learning a more practical set of tools when computational resources are limited or the dataset size exceeds a few hundred records. However, in order for it to handle large datasets, there is a huge need for efficient eigenvalue computation methods that would provide at least a good approximation of the matrix spectrum. One idea to tackle this issue would be to leverage one of the existing computation methods of the eigenvalue that rely on the evaluation of only the set of *k*-largest specified eigenvalues instead of the entire spectrum, and replacing the rest of the eigenvalues with a small constant positive value.

It is reasonable to assume that this approach yields a good approximation of the matrix spectrum due to the fact that when data dimensionality increases, the number of significant eigenvalues does not tend to grow. For high-dimensional datasets, usually only a proportion of eigenvalues is significant, and the rest can be approximated and replaced by a constant. The choice of this constant and the number of eigenvalues that would generate a good enough approximation of the spectrum and, thus, of the true von Neumann entropy, remains an open research topic.

## Figures and Tables

**Table 1:** Summary of the influence of kernel parameters on the von Neumann entropy and their relation to the model’s behavior.

$K\to I_{n}$	$K\to J_{n}$
von Neumann entropy →1	von Neumann entropy →0
σ→∞	σ→0
d→∞	d→0
overfitting	underfitting
high variance	low variance
low bias	high bias

**Table 2:** Detailed list of datasets used for experiments along with their sizes (number of rows) and dimensionalities.

Dataset	Size	Dimensionality
auto	205	26
Boston	506	14
prostate	97	10
air quality	153	6
triazines	186	59
slump	103	10
yacht	364	7
energy	768	9
mpg	404	8
student	395	33
bio	320	5
breast	198	33

**Table 3:** Correlation coefficients between the von Neumann entropy and training NMSE for the RBF kernel per dataset.

Dataset	Spearman Correlation	Pearson Correlation
auto	−0.809	−0.845
Boston	−0.925	−0.598
prostate	−0.873	−0.545
air quality	−0.883	−0.831
triazines	−0.821	−0.801
slump	−0.909	−0.551
yacht	−0.933	−0.622
energy	−0.947	−0.718
mpg	−0.951	−0.75
student	−0.939	−0.699
bio	−0.783	−0.768
breast	−0.846	−0.928

**Table 4:** Correlation coefficients between the von Neumann entropy and training NMSE for the normalized polynomial kernel per dataset.

Dataset	Spearman Correlation	Pearson Correlation
auto	−0.773	−0.75
Boston	−0.962	−0.927
prostate	−0.819	−0.758
air quality	−0.86	−0.801
triazines	−0.883	−0.795
slump	−0.809	−0.732
yacht	−0.943	−0.644
energy	−0.77	−0.691
mpg	−0.8	−0.806
student	−0.963	−0.954
bio	−0.581	−0.552
breast	−0.869	−0.841

**Table 5:** Correlation between the von Neumann entropy and training NMSE for the ELM kernel per dataset.

Dataset	Spearman Correlation	Pearson Correlation
auto	−0.652	−0.744
Boston	−0.755	−0.986
prostate	−0.82	−0.917
air quality	−0.727	−0.9
triazines	−0.593	−0.62
slump	−0.809	−0.685
yacht	−0.648	−0.935
energy	−0.745	−0.925
mpg	−0.673	−0.903
student	−0.836	−0.966
bio	−0.757	−0.909
breast	−0.396	−0.417

**Table 6:** Correlation between the von Neumann entropy and test NMSE for the RBF kernel after taking a square root and reversing the NMSE sign to the left of the minimum of the parabola.

Dataset	Spearman Correlation	Pearson Correlation
auto	0.883	0.968
Boston	0.954	0.943
prostate	0.947	0.95
air quality	0.934	0.97
triazines	0.944	0.947
slump	0.956	0.932
yacht	0.949	0.912
energy	0.957	0.943
mpg	0.942	0.960
student	0.934	0.958
bio	0.927	0.951
breast	0.892	0.976

**Table 7:** Correlation between the von Neumann entropy and test NMSE for the normalized polynomial kernel after taking a square root and reversing the NMSE sign to the left of the minimum of the parabola.

Dataset	Spearman Correlation	Pearson Correlation
auto	0.881	0.903
Boston	0.945	0.943
prostate	0.954	0.869
air quality	0.9	0.916
triazines	0.953	0.906
slump	0.938	0.877
yacht	0.926	0.927
energy	0.926	0.938
mpg	0.804	0.842
student	0.973	0.869
bio	0.876	0.884
breast	0.939	0.891

**Table 8:** Heuristic for the optimal von Neumann entropy for the RBF kernel defined as the minimum of the parabola resulting from the modeling of the test NMSE against the von Neumann entropy as a degree 2 polynomial regression.

Dataset	von Neumann Entropy
auto	0.343
Boston	0.416
prostate	0.396
air quality	0.481
triazines	0.315
slump	0.331
yacht	0.557
energy	0.413
mpg	0.471
student	0.449
bio	0.372
breast	0.389

**Table 9:** Heuristic for the optimal von Neumann entropy for the normalized polynomial kernel defined as the minimum of the parabola resulting from modeling the test NMSE against the von Neumann entropy as a degree 2 polynomial regression.

Dataset	von Neumann Entropy
auto	0.401
Boston	0.444
prostate	0.480
air quality	0.421
triazines	0.489
slump	0.413
yacht	0.463
energy	0.363
mpg	0.309
student	0.362
bio	0.439
breast	0.359

**Table 10:** Comparison of the heuristic and optimal results for the RBF kernel. From third column onwards: heuristic σ, heuristic vNe, optimal NMSE, heuristic NMSE, optimal number of tries for σ, heuristic number of tries for σ.

Data	σ	heu.σ	heu.vNe	opt.nmse	heu.nmse	opt.#σtried	heu.#σtried
auto	0.3	8.554	0.451	0.905	0.906	24	5
Boston	6	5.669	0.468	0.174	0.176	24	7
prostate	0.1	1	0.418	0.143	0.194	24	1
air quality	3	5.669	0.439	0.559	0.618	24	7
triazines	0.1	1	0.442	0.851	0.879	24	1
slump	0.3	3.885	0.449	0.136	0.315	24	9
yacht	66	33	0.435	0.012	0.015	24	2
energy	6	13.223	0.459	0.082	0.086	24	3
mpg	33	33	0.457	0.213	0.213	24	2
student	0.06	2.783	0.409	0.219	0.233	24	11
bio	3	8.554	0.453	0.807	0.796	24	5
breast	0.3	3.885	0.444	0.879	0.897	24	9
**average**			0.444	0.415	0.442	24	5.17

**Table 11:** Comparison of the heuristic and optimal results for the normalized polynomial kernel.

Data	*d*	heu. *d*	heu.vNe	opt.nmse	heu.nmse	opt.# *d*tried	heu.# *d*tried
auto	50	31.175	0.433	0.878	0.913	46	3
Boston	25	19.649	0.442	0.186	0.188	46	5
prostate	1	3.721	0.410	0.128	0.194	46	13
air quality	9	19.649	0.41	0.582	0.616	46	5
triazines	2	3.721	0.44	0.858	0.899	46	3
slump	2	12.526	0.405	0.127	0.269	46	7
yacht	200	80	0.361	0.012	0.02	46	2
energy	20	31.175	0.364	0.08	0.085	46	3
mpg	60	80	0.404	0.206	0.208	46	2
student	3	12.526	0.444	0.223	0.242	46	7
bio	50	31.175	0.435	0.777	0.795	46	3
breast	4	12.526	0.392	0.853	0.898	46	7
**average**			0.412	0.41	0.444	46	5

**Table 12:** Correlation between the von Neumann entropy and training NMSE for the RBF kernel and normalized polynomial kernel for the Abalone dataset.

Dataset	Kernel	Spearman Correlation	Pearson Correlation
abalone	RBF	−0.986	−0.926
abalone	normalized polynomial	−0.889	−0.954

**Table 13:** Correlation between the von Neumann entropy and test NMSE for the RBF kernel and normalized polynomial kernel for the Abalone dataset.

Dataset	Kernel	Spearman Correlation	Pearson Correlation
abalone	RBF	0.98	0.94
abalone	normalized polynomial	0.90	0.78

**Table 14:** Comparison of the RBF kernel optimal results with the heuristic ones for the Abalone dataset.

Dataset	σ	heu.σ	heu.vNe	opt.nmse	heu.nmse	opt.#σtried	heu.#σtried
abalone	10	33	0.406	0.422	0.44	16	2

**Table 15:** Comparison of the normalized polynomial kernel optimal results with the heuristic ones for the Abalone dataset.

Dataset	*d*	heu.d	heu.vNe	opt.nmse	heu.nmse	opt.#*d*tried	heu.#*d*tried
abalone	50	70	0.32	0.42	0.443	32	2

## Data Availability

Not applicable.
